# Nursing Services in the First Level of Care in Colombia. Analysis of the Offer 2002-2020*

**DOI:** 10.17533/udea.iee.v40n3e04

**Published:** 2023-02-09

**Authors:** Genny Paola Fuentes Bermudez, Oneys del Carmen De Arco Canoles

**Affiliations:** 1 Nurse, Master’s, PhD (c). Professor, Universidad Nacional de Colombia. Colombia. Email: gfuentesb@unal.edu.co Universidad Nacional de Colombia Universidad Nacional de Colombia Colombia gfuentesb@unal.edu.co; 2 Nurse, Master’s, Professor, Universidad Nacional de Colombia. Colombia. Email: ocdec@unal.edu.co Universidad Nacional de Colombia Universidad Nacional de Colombia Colombia ocdec@unal.edu.co

**Keywords:** nursing services, primary health care, health services accessibility, law of supply and demand, nursing administration research, primary care nursing., servicios de enfermería, atención primaria de salud, accesibilidad a los servicios de salud, ley de la oferta y la demanda, investigación en administración de enfermería, enfermería de atención primaria., serviços de enfermagem, atenção primária à saúde, acesso aos serviços de saúde, lei da oferta e da procura, pesquisa em administração de enfermagem, enfermagem de atenção primária.

## Abstract

**Objective.:**

This work sought to characterize the primary care nursing consultation services reported in the official systems of health services records in Colombia between 2002 and 2020.

**Methods.:**

This was a descriptive, cross-sectional, retrospective study. Node geographic analysis and descriptive statistics were performed for quantitative data from the Special Registry of Health Providers and the Ministry of Health and Social Protection.

**Results.:**

The study identified 6079 nursing services of which 72% are outpatient, 95.05% are assigned to institutions providing health services, 99.75% are of low complexity, and 48.22% of the offer was created in the last five years. The nodes with the highest increase in the offer of services are Caribbean (*n* = 909) and Pacific (*n* = 499), while Amazon (*n* = 48) showed the lowest offer in the last five years.

**Conclusions.:**

Disparity is evident in the availability of services by region and node, in addition to a low liberal exercise to provide nursing care.

## Resumen

**Objetivo.:**

Caracterizar los servicios de consulta de enfermería de primer nivel de atención reportados en los sistemas oficiales de registros de servicios de salud en Colombia durante el periodo 2002-2020.

**Métodos.:**

Estudio descriptivo, transversal de corte retrospectivo. Se realizó análisis geográfico por nodos y estadística descriptiva para los datos procedentes del Registro Especial de Prestadores en Salud (REPS) del Ministerio de Salud y Protección Social.

**Resultados.:**

Se identificaron 6079 servicios de enfermería de los cuales el 72% son ambulatorios, el 95.05% están adscritos a instituciones prestadoras de servicios de salud, un 99.75% son de complejidad baja y, el 48.22% de la oferta se creó en los últimos 5 años. Los nodos que han mostrado mayor aumento en la oferta de servicios son Caribe (*n*=909) y Pacífico (*n*=499), mientras que Amazonia (*n*=48) ha mostrado la menor oferta en los últimos cinco años.

**Conclusión.:**

Existe disparidad en la disponibilidad de servicios por región y nodo, además de un bajo ejercicio independiente para la provisión de atención en enfermería.

## Resumo

**Objetivo.:**

Caracterizar os serviços de consulta de enfermagem de primeiro nível de atenção informados nos sistemas oficiais de registro dos serviços de saúde na Colômbia durante o período 2002-2020.

**Métodos.:**

Estudo descritivo, transversal, retrospectivo. Foi realizada análise geográfica por nós e estatística descritiva para os dados do Cadastro Especial de Provedores de Saúde (REPS) do Ministério da Saúde e Proteção Social.

**Resultados.:**

Foram identificados 6079 serviços de enfermagem, sendo 72% ambulatorial, 95.05% vinculados a instituições que prestam serviços de saúde, 99.75% são de baixa complexidade e 48.22% da oferta foi criada nos últimos 5 anos. Os nós que apresentaram maior aumento na oferta de serviços são Caribe (*n* = 909) e Pacífico (*n* = 499), enquanto a Amazônia (*n* = 48) apresentou a menor oferta nos últimos cinco anos.

**Conclusão.:**

Há disparidade na disponibilidade de serviços por região e nó, além de um baixo exercício independente para a prestação de cuidados de enfermagem

## Introduction

The aim of the primary health care approach is to maximize the level and distribution of health and wellbeing through the integrated articulation of services of the first level of care and public health, multi-sector construction of public policies and the call to action, and empowerment of people and communities.(1) Primary care has been recognized as the nuclear and central axis of health systems, as well as the social and economic development of communities. According to the Alma-Ata Declaration, it represents the first level of contact human beings have at individual and collective level with health systems, besides favoring access to health care in different scenarios in which the person develops.(2) In 2020, the operational framework for primary health care by the World Health Organization (WHO) ratified that in addition to its being the first contact, it is configured as a key process to provide accessible, continuous, comprehensive, and patient-centered care.(1)

Achieving universal coverage implies, in primary-care oriented health systems, availability of technologies that facilitate access to health services, efficiency in using health resources to facilitate financial sustainability, and availability of professionals able to respond to the needs of the population in any of the levels of care through the development of the staff’s knowledge and skills, and the guarantee of dignified, decent, and safe working conditions.(3) Given the current approach on primary health care, proposed by the Astana Declaration and implemented within the operational framework developed by the WHO(1), it becomes necessary to identify the resources and capacities of the national health systems within the primary care component. Participation by Nursing in these services contributes to improving the quality of life, greater adherence to treatment, higher level of knowledge, and higher rates of patient satisfaction. (4)

Nursing services encompass autonomous care and in collaboration dispensed to people of all ages, families, groups and communities, sick or not, and under all circumstances.(5) It comprises health promotion, disease prevention, and care dispensed to the sick, handicapped, and individuals with terminal illnesses.(5,6) Within the framework of the policy on comprehensive health care for Colombia, nursing activities in primary care, commonly defined as nursing consultations, have centered on vaccinations, evaluation of growth and physical, motor, cognitive and socio-emotional development, assessment of the nutritional status, assessment of sexual and reproductive health, and detection of alterations in all the phases of the vital cycle;(7) however, it is also possible to identify interventions around education and technical care, like administration of medications or management of complex wounds.(8) The country’s regulations on the creation of health services has included the qualification of nursing services in outpatient modality, home care, and mobile units, with the special registry of health services providers being the official source to have access to information related with the availability of health services at the national level.(9) 

Regulatory changes in the Colombian health system reaffirm the strategic role of the first level of care and the importance of the nursing consultation in these services, becoming necessary to characterize its development to determine consolidation strategies and establish actions that permit increasing its capacity and coverage. The use of national health data, collected by the Colombian Ministry of Health and Social Protection since 2002, (10) is an underused decision-making tool in the country, hence, its analysis will permit management based on data and with it, the transformation of information in health results that improve the quality of life of the population. Bearing in mind the aforementioned, the aim of the study was to characterize nursing services of the first level of care reported in official systems of health services records in Colombia between 2002 and 2020.

## Methods

This was a descriptive, retrospective cross-sectional study of the offer of nursing services enabled in the Colombian territory. Data related with the offer, modality of care, complexity, provider and year of creation of the nursing services were taken from official sources, like the Special Registry of Health Providers (REPS, for the term in Spanish) by the Ministry of Health and Social Protection. (10) Information on the population per department to calculate the density of services was obtained from the 2018 National Population and Housing Census.(11)

Using the REPS, nursing services were characterized in Colombia by using five variables: a) number of services; b) modality of nursing care; c) type of practice (autonomous or linked to an organization); d) level of complexity classified as low, medium, and high; and e) evolution over time and growth of services.

Colombia has a vast geographic and population diversity; it is divided into 32 departments and one capital district (Bogotá D.C.). The geographic analysis used the proposal by León *et al*., which consists in grouping the 33 regions of the country into seven nodes(12) denominated: Amazon (Putumayo, Amazonas, Caquetá, Guaviare, Vaupés, and Guainía); Orinoquía (Meta, Vichada, Casanare, Arauca, and Cundinamarca); Northeast (Boyacá, Santander, Norte de Santander, and Cesar), Pacific (Nariño, Cauca, Valle del Cauca, and Chocó); Central (Antioquia, Caldas, Quindío, Risaralda, Tolima, and Huila); Caribbean (La Guajira, Magdalena, Atlántico, Bolívar, Sucre, Córdoba, San Andrés); and Bogotá (Bogotá D.C.) to standardize the presentation of results in regions (departments and capital district) with common sociodemographic characteristics. 

Absolute and relative frequency measures were used for the variables care setting, level of complexity, temporary evolution and growth of services, and type of provider to characterize nursing services per region. Descriptive statistics analysis was used for density of the number of services per 100-thousand inhabitants, grouping the results into quartiles to categorize the availability of services per region. Measures of central tendency were used to present information on the five variables for each of the geographic nodes in Colombia.

## Results

### Number of nursing services

The study identified 6,076 nursing services. The Caribbean node groups the territories with greater number of nursing services (*n* = 1,673), followed by the Pacific node (*n* = 1212) and the Northeast node (*n* = 1064), while the Amazon node has the lowest number of services. The regions with the highest absolute registry of services are Valle del Cauca (*n* = 576), Bogotá (*n* = 98), and Santander (*n* = 411); 59.52% of the services in the country are concentrated in 10 regions, which are Valle del Cauca, Bogotá D.C, Santander, Bolívar, Cundinamarca, Atlántico, Nariño, Córdoba, Magdalena, and Boyacá; moreover, great disparity is observed in the offer of services among regions, for example: Valle del Cauca has 576 services unlike Vaupés and Guainía with five services each (Table 1).

**Table 1:** Absolute frequencies of the study variables: number of services, care setting, and type of provider. Colombia, 2002-2020

Node	Region	Services		Modality of care*			Type of practice		Region
		Number	Percentage	Home	Outpatient	Mobile unit	Institutional	Independent professional	
Amazonia	Caquetá	44	0.72	12	42	9	44	0	Caquetá
	Putumayo	41	0.67	19	40	8	41	0	Putumayo
	Amazonas	14	0.23	1	14	1	14	0	Amazonas
	Guaviare	9	0.14	5	9	1	9	0	Guaviare
	Guainía	5	0.09	0	5	0	5	0	Guainía
	Vaupés	5	0.09	0	5	3	5	0	Vaupés
	Node total	118	1.94%	37	118	22	118	0	Node total
Orinoquía	Cundinamarca	329	5.41	70	283	18	326	3	Cundinamarca
	Meta	180	2.96	59	145	8	172	8	Meta
	Casanare	69	1.14	36	58	5	69	0	Casanare
	Arauca	56	0.92	34	37	3	56	0	Arauca
	Vichada	11	0.18	1	10	2	11	0	Vichada
	Node total	645	10.61%	200	533	36	634	11	Node total
Northeast	Santander	411	6.76	141	357	13	409	2	Santander
	Boyacá	248	4.09	45	235	19	241	7	Boyacá
	Cesar	216	3.55	51	175	16	216	0	Cesar
	Norte de Santander	189	3.12	38	162	7	187	2	Norte de Santander
	Node total	1,064	17.52%	275	929	55	1,063	11	Node total
Central	Antioquia	293	4.8	87	251	9	278	15	Antioquia
	Tolima	167	2.74	57	133	21	165	2	Tolima
	Huila	124	2.04	39	113	11	122	2	Huila
	Risaralda	110	1.82	22	101	12	110	0	Risaralda
	Caldas	103	1.7	19	99	8	103	0	Caldas
	Quindío	69	1.15	18	65	5	67	2	Quindío
	Node total	866	14.25%	242	762	66	845	21	Node total
Caribbean	Bolívar	363	5.97	108	330	20	360	3	Bolívar
	Atlántico	327	5.38	81	293	10	327	0	Atlántico
	Córdoba	279	4.59	45	263	6	279	0	Córdoba
	Magdalena	268	4.42	81	242	25	268	0	Magdalena
	Sucre	224	3.68	58	211	17	223	1	Sucre
	La Guajira	203	3.34	66	187	30	203	0	La Guajira
	San Andrés and Providencia	9	0.15	2	7	0	8	1	San Andrés and Providencia
	Node total	1,673	27.53%	441	1533	108	1,776	5	Node total
Pacific	Valle del Cauca	576	9.47	105	534	26	574	2	Valle del Cauca
	Nariño	318	5.23	118	283	38	314	4	Nariño
	Cauca	166	2.74	32	156	7	165	1	Cauca
	Chocó	152	2.51	40	123	83	152	0	Chocó
	Node total	1,212	19.95%	295	1,391	154	1,205	7	Node total
Bogotá	Bogotá D.C	498	8.20%	147	394	7	460	38	Bogotá D.C
National Total		6,076	100%	1,637	5,660	448	6,101	93	

**Map 1:**
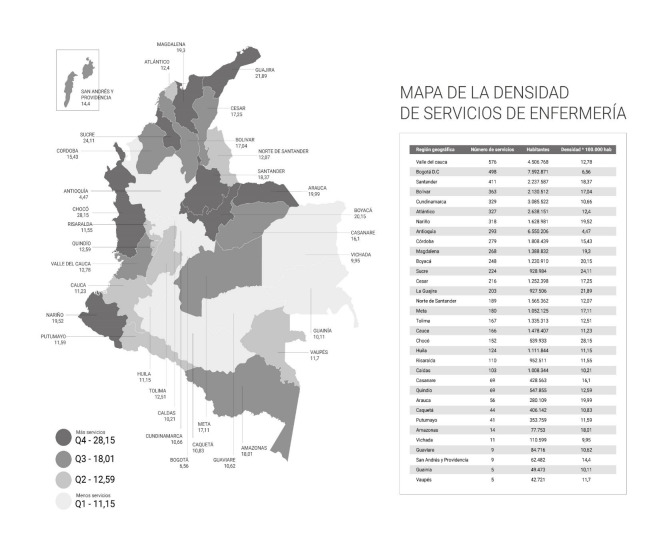
Density of nursing services per 100,000 inhabitants. Colombia 2002-2020

The national density mean is 14.53 services per 100-thousand inhabitants. The region with greatest density of services is Chocó (28.15 per 100-thousand), followed by Sucre (24.11 per 100-thousand), and La Guajira (21.89 per 100-thousand), while those with the lowest density are Antioquia and Bogotá DC (Map 1). The density per nodes observed is Pacific with 17.92, Caribbean with 17.79 services per 100-thousand inhabitants, Northeast with 16.96 services per 100-thousand inhabitants, Orinoquía with 14.76 services per 100-thousand inhabitants, Amazonia with 12.14 services per 100-thousand inhabitants, Central with 10.41 services per 100-thousand inhabitants, and Bogotá with 6.56 services per 100-thousand inhabitants.

### Nursing care modality

The nursing care modality corresponds to outpatient services (72%), home services (21%), and mobile unit (6%). In all, there are 7447 services because the same service can be enabled in several modalities. The nodes with highest offer of services in the three modalities are Caribbean (n = 1533 outpatient; n = 441 home; n = 108 mobile units) and Pacific (*n* = 1,096 outpatient; *n* = 295 home; *n* = 154 mobile units). The Amazon node has the lowest gross number of services in home modality (n = 37) and outpatient (n = 115), while the lowest number of services in mobile unit modality are found in the Bogotá node (*n* = 7).

The mean for home services is 49.60 per region and the offer is found mainly in the regions of Bogotá (*n* = 147), Santander (*n* = 41), Nariño (*n* = 118), Bolívar (*n* = 108), and Valle del Cauca (*n* = 105). The mean for outpatient services is 162.48 services per region and 45.45% of the regions is above the national mean, with Valle del Cauca (*n* = 534), Bogotá D.C (*n* = 394), and Santander (*n* = 357) having the highest number of services in this modality. The mean of nursing mobile units is 13.57 services per region, with Chocó quite above with 83 services, followed by Nariño with 38 services (Table 1). 

### Type of nursing practice

The provision of nursing services in primary care is linked to health institutions by 95.05% (*n* = 5775), of which 3624 are private organizations. Also noted is that 3.42% of the institutions have a social object different from providing health services (*n* = 208), principally educational institutions, centers for the elderly, and rehabilitation centers. Enabling nursing services as independent professionals is low with 1.53%, which corresponds to 93 nursing services (Table 1). The nursing practice linked to institutions has the highest absolute frequencies in the Caribbean node (*n* = 1668), followed by the Pacific node (*n* = 1205), while the autonomous practice is observed with greater frequency in the Bogotá node with 38 services. The Amazon node reports the lowest number of nursing services associated with organizational practice (*n* = 118) and autonomous practice (*n* = 0). In the regional analysis, Bogotá DC concentrates the highest number of nursing services linked to an organization (*n* = 460) and derived from an autonomous practice (*n* = 38). The lowest figures are shown in Guainía and Vaupés with five institutional nursing services and none for independent practice (Table 1).

### Complexity of care

The study identified 6064 low-complexity nursing services (99.81%), 11 of medium complexity, and one of high complexity. The low-complexity services are distributed in the Caribbean node (n = 1669), the Pacific node (*n* = 1210), the Central node (*n* = 862), the Northeast node (*n* = 1063), Orinoquía (*n* = 645), Amazon (n = 118), and Bogotá (*n* = 498). The medium-complexity services are dispersed among the Caribbean node (*n* = 5), Central node (*n* = 4), and Pacific node (*n* = 2). The only high-complexity service reported belongs to the Northeast node (Santander). The regions with the medium-complexity services are Atlántico (*n* = 3), Antioquia (*n* = 2), Caldas (*n* = 2), Chocó (*n* = 1), Guajira (*n* = 1), Magdalena (*n* = 1), and Valle del Cauca (*n* = 1). 

### Time evolution and growth of services

It was found that 48.22% (*n* = 2930) of the aperture of services has been concentrated in the last five years (2016-2020). With 2020 being the year with greatest openings with 782 new services, followed by 2006 with 691 (Graphic 1). The record shows 10 services that do not report aperture date, thus, these were not considered to analyze this variable.

**Graphic 1:**
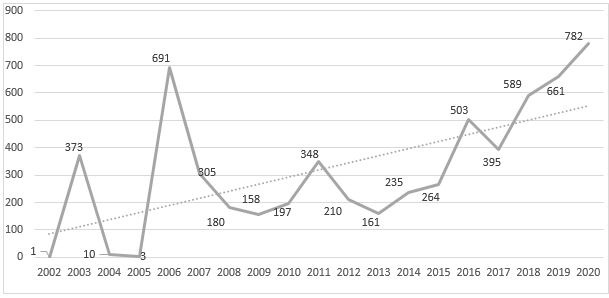
Overall growth of nursing services. Colombia, 2002-2020

Between 2016 and 2020, the nodes that have shown greater increase in the service offer are Caribbean (n = 909) and Pacific (n = 499), while Amazon (n = 48) has revealed the lowest offer during the last five years. 

## Discussion

The current approach on primary health care for universal coverage and compliance of the goals of sustainable development related with health propose as indicator assessment of the density of health workers per population, disaggregating the level where services are provided and the subnational area where the resource is available.(13) Following this recommendation, the work present results on the services of first level of nursing care in the Colombian territory, evaluating its availability per regions and nodes to understand the geographic and population differences. 

The national mean of density of nursing services per 100 000 inhabitants is 14.53, a low figure when compared with data from member countries of the European Union with an availability of 44 units of primary care per 100 000 inhabitants.(14) It is necessary to increase the offer of nursing services, given that they enhance access for the population to primary-care services and speed up the progress toward universal coverage. Although the Global Health Observatory reports data related with the density of nurses per every 10,000 inhabitants, as well as the density of nurses working in mental health per every 100 000 inhabitants, no figures have been published on the availability of nursing services, which supposes a void in the design of indicators and, hence, the lack of a standard that permits comparing the results obtained in this study. (15)

The density of nursing services in regions, like Antioquia (4.47) and Bogotá (6.56) has the lowest levels; however, these territories have offers of other health services, for example, these two regions have 940 and 1,650 health-service provider institutions, respectively, which allows them meet the demands of the population.(16) The Pacific node has the highest density mean per 100 000 inhabitants; however, the regions with highest number of nursing services are Bogotá, Antioquia, and Valle del Cauca, although the results of the density indicator are at suboptimal levels; the foregoing related with the high population demand. It is relevant to highlight that the use and analysis of indicators, like density of services per 100 000 inhabitants, when considering the number of inhabitants per region, may lead to an erroneous interpretation of the offer of nursing services. Regarding the known regional heterogeneity and with the purpose of avoiding confusing conclusions, regional raw data are presented concomitantly in this study.

This study found important disparity in the offer of services among regions (Valle del Cauca has 576 services, unlike Vaupés and Guainía with five services each). These results are coherent with global research that shows a greater concentration of health professionals in urban areas or large cities;(17-19) for Colombia, this is similar to that found by Mendieta and Jaramillo, whose research indicates that the country continues being incredibly unequal regarding access by the population to hospital centers and health professionals, given that although Colombia has 23 of the 58 best hospitals in Latin America, these centers are concentrated in Bogotá, Medellín, Cali, and Bucaramanga, while regions, like Orinoquía, Pacific, or Amazon do not have a basic health center.(20) 

Nursing services are framed principally on providing outpatient care (72%); nevertheless, the study detected 21% home services, which implies the professional and disciplinary response to the demographic and epidemiological changes derived from the chronicity of long-standing non-communicable diseases, (21) as well as mobile nursing services as a mechanism that permits bringing health services closer to the communities. It was identified that the nursing practice is carried out in institutions with social object different from that of providing health services in 3.42% (*n* = 208), like educational institutions, centers for the elderly, and rehabilitation centers, among others. This result reflects the participation by nursing professionals in different settings where the individuals develop their work and ratifies their intervention in the different stages of the vital cycle. Nursing care in educational centers permits contact by the population with health services without being within the framework of health institutions, besides favoring the construction of physically (22) and mentally(23,24) healthy environments, as well as the possibility of extending the interventions to the family (25).

Enabling nursing services as professionals who perform a liberal exercise(26) is at 1.53%, corresponding to 93 nursing services, which is similar to other professionals in medicine and psychology, but extremely low when considering that in the country 652 of the therapy professionals perform this type of practice in home service modality.(10) Although nursing services can enhance the population’s access to services of first level of care, it is necessary to improve the number of professionals in the region (27,28); according to the Organization for Economic Co-operation and Development (29) for 2019, in Colombia, the availability of nursing staff was 1.3 per 1,000 inhabitants, only above Indonesia with 1.2 and far below the European nations. 

Colombian regulations recognize that the offer of services can be classified by their complexity, as low and medium, with the latter for professionals with Specialist degree.(9) This study identified 10 medium-complexity services, which is a significant finding in function of the offer of 15 disciplinary graduate training programs in the areas of child, maternal-perinatal, elderly and family nursing.(30) The study found 48.22% (*n* = 2,930) of the aperture of services has concentrated in the last five years (2016 - 2020). This growth responds to the WHO call to enhance primary health care, to achieving the objectives of sustainable development,(31,32) particularly health and wellbeing, and decent work and economic growth, as pillars that directly affect the health system. Nursing services of first level of care are, then, a response regarding the imminent need to increase the capacity of health systems and meet the demand for primary care from the people, families, and communities. (33)

Studies have been conducted on the distribution of the labor force in nursing or the availability of professionals in the regions, (34-36) however, given that the professional nursing practice is present in hospital environments, it is considered that a future area of research should focus on the distribution of nursing services in the first level of care, on their relation with improving the quality of life of the population and the population’s health results, and incorporation of advanced nursing care in services of first level of care in Colombia. 

In conclusion, the characterization of nursing services identified an increase in the offer of nursing services and their availability throughout the Colombian territory; however, the number of services available has important differences per region and per node, which implies lower opportunities for access to health care. Nursing services are provided mainly in outpatient manner and linked to health organizations, thereby, home nursing care and their presence in educational settings represent a strategy to bring health services closer to the population.
